# Flavorless vs. Flavored Electronic Cigarette-Generated Aerosol and E-Liquid on the Growth of Common Oral Commensal Streptococci

**DOI:** 10.3389/fphys.2020.585416

**Published:** 2020-11-23

**Authors:** Jacob S. Fischman, Swapna Sista, DongKeun Lee, Giancarlo A. Cuadra, Dominic L. Palazzolo

**Affiliations:** ^1^Department of Biology, Muhlenberg College, Allentown, PA, United States; ^2^Department of Physiology, DeBusk College of Osteopathic Medicine, Lincoln Memorial University, Harrogate, TN, United States

**Keywords:** ECIG, E-liquid flavors, aerosol, oral commensal bacteria, toxicity, bacterial growth

## Abstract

**Introduction:**

Electronic cigarette (ECIG) use or vaping has become popular globally. While the question “Is vaping safer than smoking?” continues, it is becoming clearer that one of the most dangerous components of E-liquids are the flavorings. Since the oral cavity is the first anatomical site to be assaulted by ECIG aerosol, the aim of this study is to test the hypothesis that flavored ECIG aerosols or E-liquids pose a more detrimental effect on the growth of commensal oral streptococcal bacteria compared to flavorless aerosols or E-liquids.

**Methods:**

Kirby Bauer assays and 24-h planktonic growth curves were used to compare the effects of flavorless vs. flavored (tobacco, menthol, cinnamon, strawberry and blueberry) ECIG-generated aerosols and E-liquids on the growth of four common strains of oral commensal bacteria (*Streptococcus gordonii*, *Streptococcus intermedius*, *Streptococcus mitis* and *Streptococcus oralis*).

**Results:**

Kirby Bauer assays revealed inhibition of growth for all bacteria tested when exposed to 100% menthol, cinnamon or strawberry flavors. In contrast, 5% flavor in E-liquid had no effect. When exposed to 100 puffs of ECIG-generated aerosol ± flavors (≈ 0.05% flavor in brain heart infusion media) or an equivalent amount of E-liquid ± flavors, twenty-four hour planktonic growth curves indicated no effect on growth for all streptococci tested. Subsequent twenty-four hour planktonic growth curves testing the effects of E-liquid ± flavors (0.0625, 0.125, 0.25, 0.3125, 0.625, and 1.25% flavor in brain heart infusion media) revealed dose-dependent inhibition of growth, particularly for menthol, cinnamon and strawberry), for all bacteria tested.

**Conclusion:**

These results support the hypothesis that flavored E-liquids are more detrimental to the growth of oral commensal bacteria than unflavored E-liquids. The streptococci tested in this study are early colonizers and part of the foundation of oral biofilms and dental plaque. Disturbances in the composition and growth of these primary colonizers is crucial to the development of a healthy dental plaque and host-bacteria interactions. E-liquids and their aerosols containing flavoring agents alter the growth of these bacteria. Such perturbations of pioneering oral communities pose a potential risk to the health of the oral cavity and, ultimately, health in general.

## Introduction

Electronic Cigarettes (ECIG) are devices which aerosolize a liquid (E-liquid) which is subsequently inhaled as one would inhale smoke from a traditional cigarette. In its liquid state, E-Liquid is comprised primarily of propylene glycol and/or vegetable glycerine as the base humectants, nicotine and any number of flavoring agents. The E-liquid contains dissolved nicotine in concentrations ranging from 0 mg/mL to 24 mg/mL (or higher). Consequently, ECIG devices have become a popular surrogate for smoking as a means to satiate nicotine dependence with what many believe to be a safer, healthier and trendier alternative to cigarettes. While it is recognized that vaping is not completely safe, some scientists and healthcare professionals (Farsalinos and Polosa, 2014; Farsalinos and Gillman, 2018; Stephens, 2018; St Helen et al., 2020) report that inhaling aerosolized E-liquids has the potential to induce fewer health-related complications than inhaling traditional cigarette smoke based on the fact that E-liquids contain fewer and less harmful substances (particularly those substance deemed carcinogenic) than combusted tobacco. For example, there are far more carcinogenic compounds in tobacco smoke, including specific N-nitrosamines, polycyclic aromatic compounds, volatile organic compounds and carcinogenic heavy metals (Talhout et al., 2011) than in E-liquid aerosol (Palazzolo, 2013; Farsalinos and Polosa, 2014; Farsalinos and Gillman, 2018; Stephens, 2018; St Helen et al., 2020). Alarmingly, there have been many recent reports involving lung injuries caused by E-liquid aerosol (Chand et al., 2019). However, these injuries are often associated with substances such as tetrahydrocannabinol (THC) and cannabidiol (CBD) oils, many of which are illegally obtained from black markets (Kalininskiy et al., 2019; Conuel et al., 2020; Duffy et al., 2020). In addition, flavoring compounds such as cinnamaldehyde induce inflammation and cytotoxicity in airway tissues (Bahl et al., 2012; Muthumalage et al., 2018). Given that ECIGs have been around for only a relatively short period of time, others agree that not enough is known about the long-term health consequences that ECIG-generated aerosols may manifest in users (Löhler and Wollenberg, 2019), including the possibility of latent ECIG-induced carcinogenicity. Current data suggest that vaping ECIGs has become more prevalent, especially among teens. For example, studies performed by the Centers for Disease Control (CDC) found that ECIG usage among high school students rose from 1.5%, in 2011 to 27.5% in 2019 (Jamal et al., 2017, 2011–2016; Center for Disease Control and Prevention, 2019a). Most recently, however, the CDC (2020) found that ECIG usage among high school students to decrease to 19.6%. This decrease most likely reflects state bans (As the Number of Vaping-Related Deaths Climbs, These States Have Implemented E-Cigarette Bans, 2019) on ECIG devices, particularly those containing flavored E-liquids, as a consequence of public disquiet concerning the many vaping-related injuries reported in 2019 (Chand et al., 2019; Kalininskiy et al., 2019; Conuel et al., 2020; Duffy et al., 2020).

More troubling is that all nicotine use rates (from both ECIG and tobacco products) have risen to as high as 31.2% among high school students and 12.5% among middle school students between 2011 and 2019 (Center for Disease Control and Prevention, 2019a). These statistics demonstrate a marked increase compared to the 2016 data that showed nicotine usage among middle and high school students to be 7% and 20%, respectively (Jamal et al., 2017). The introduction of newer and more appealing flavored E-liquids, as well as innovations such as easily concealable Juul sticks, are factors contributing to the increased nicotine use rate among teens in the United States (Krüsemann et al., 2019; Vogel et al., 2018). Since E-liquid components, including flavoring agents^1^ are readily available for purchase online, this allows users to make their own E-liquid mixtures, in any proportions they choose, prior to vaping. Such freedom and “do it yourself” approach to vaping allows for extreme contents of flavors and other illicit constituents in inhaled aerosols, exacerbating the potential to develop vaping-related injuries and hospitalizations (Center for Disease Control and Prevention, 2019b; Fonseca Fuentes et al., 2019). In contrast to the decreasing nicotine usage from cigarettes among teens observed throughout the early 2000’s, nicotine usage is returning to levels not seen since the height of smoking popularity in the mid 1970’s; and many attribute this to a meteoric rise in ECIG popularity (Pampel and Aguilar, 2008; Center for Disease Control and Prevention, 2019a).

Cigarette smoking is known to have serious harmful effects on the oral microbiota and the oral cavity itself, specifically by disrupting the delicate balance between the microbes and the host. The normal oral microbiota is composed of numerous commensal and pathogenic bacterial species that form intricately organized polymicrobial communities on oral surfaces (Kolenbrander, 2000; Diaz et al., 2006; Kolenbrander et al., 2006). These microbes exist in a homeostatic state, with each other and with the host, as multi-species biofilms in the mouth. However, their growth can be individually modeled planktonically in liquid cultures (Aas et al., 2005; Marsh et al., 2015; Samaranayake and Matsubara, 2017; Kilian, 2018). Common commensal species include *Streptococcus gordonii*, *Streptococcus intermedius*, *Streptococcus mitis* and *Streptococcus oralis* (Garnier et al., 1997; Jenkinson and Lamont, 1997; Rosan and Lamont, 2000; Aas et al., 2005; Kolenbrander et al., 2006; Colombo et al., 2007). These commensal species live in a symbiotic relationship with their human hosts, competitively antagonizing the growth of pathogenic microbes (Kreth et al., 2008; Avila et al., 2009; Gross et al., 2012; Herrero et al., 2016). These four species are among the first to colonize oral surfaces and serve as a scaffold for other oral microbes, thus leading to the growth of multi-species biofilms (Socransky et al., 1998; Gross et al., 2012; Teles et al., 2012). These species also serve a beneficial role to the human host in the prevention of both caries and periodontal disease (Hasegawa et al., 2007; Gross et al., 2012; Herrero et al., 2016; Huang et al., 2018; Liu et al., 2018; Thurnheer and Belibasakis, 2018). For example, *S. gordonii* and *S. intermedius* have been shown to reduce invasion of the periodontal pathogen, *Porphyromonas gingivalis*, into oral epithelial cells, and may protect against gingivitis (Hanel et al., 2020). Oral health and overall systemic health are intrinsically linked. For example, several studies link *P. gingivalis* to diseases outside of the oral cavity such as diabetes, cardiovascular diseases and even Alzheimer’s disease (Mealey, 1999; Seymour et al., 2007; Amano and Inaba, 2012; Borgnakke et al., 2013; Dominy et al., 2019). Similarly, several species of oral streptococci, including *S. gordonii*, *S. mitis, S. sanguinis* and *S. oralis* are considered commensals within the oral cavity, but also implicated in infective endocarditis (Abranches et al., 2018). Therefore, any adverse activity suffered in the oral cavity due to ECIG-generated aerosol exposure has the potential to lead to both oral and systemic disease (Holmlund et al., 2017).

Smoking tobacco is the top contributor to periodontal disease, doubling the chances to develop the condition (Palmer et al., 2005; Kanmaz et al., 2019). Cigarette smoke has been demonstrated to disrupt the formation of healthy oral biofilms by promoting and recruiting pathogenic bacteria such *Fusobacterium, Fretibacterium, Corynebacterium, Cardiobacterium, Filifactor, Synergistes*, and *Selenomonas*, along with respiratory pathogens *Haemophilus* and *Pseudomonas* during the early formation of dental plaque (Kumar et al., 2011; Moon et al., 2015; Rodríguez-Rabassa et al., 2018). Mechanistically, metatranscriptomic and proteomic analysis reveals that oral commensal bacteria downregulate metabolic genes while pathogens thrive under the same conditions by upregulating virulence genes such as lipopolysaccharides, flagella and capsule; thus gaining space and resources over commensal streptococci (Shah et al., 2017). Such perturbations were reported to promote increased gingivitis (Löe and Silness, 1963; Kumar et al., 2011). Cigarette smoke modulates the oral microbiota by affecting salivary cytokine content. For example, smokers were observed to have upregulated expression of IL-2, IL-4 and adrenocorticotropic hormone and downregulated expression of MDC (n-[2-(1-maleimidyl)ethyl]-7-diethylaminocoumarin-3-carboxamide), IL-5, IL-7, IL-10, insulin and leptin compared to non-smokers (Rodríguez-Rabassa et al., 2018). Furthermore, IL-2 and IL-4 upregulation suggests activation of an immune response (Rodríguez-Rabassa et al., 2018). As recently described by Kumar and coworkers, E-liquids and their aerosols have also been shown to confer negative effects (Kumar et al., 2019). For example, antimicrobials lysozyme and immunoglobulin A are significantly decreased in the saliva of ECIG users (Cichoñska et al., 2019) as well as a pronounced adherence and biofilm growth of cariogenic pathogen *Streptococcus mutans* (Kim et al., 2018). Some data even suggest that ECIG-generated aerosol may be as dangerous (or potentially more dangerous) than conventional smoking (Jensen et al., 2015; Holliday et al., 2016; Yu et al., 2016).

Many studies have been performed to evaluate the safety of E-liquids and/or their aerosols on lung tissue and bronchial epithelial cells; however, studies concerning the oral microbiota are limited. E-liquids have demonstrated pro-inflammatory effects in human monocytes, and display toxic effects on human stem cells as well as terminally differentiated human cells (Bahl et al., 2012; Muthumalage et al., 2018; Pushalkar et al., 2020). Among the pulmonary tissue studies, research supports that flavoring agents found in cinnamon, strawberry, blueberry, menthol and tobacco, and not the base humectants (i.e., propylene glycol and/or vegetable glycerin) are responsible for cytokine production and adverse effects such as cell death (Leigh et al., 2016, 2018; Sundar et al., 2016). Currently, ECIG studies primarily focus on airway tissues. Little information is available concerning the effects of ECIG-generated aerosol on the oral cavity and even less is known about the effects on the oral microbiota. In one study (Cichoñska et al., 2019), ECIG users were observed to have diminished levels of oral lysozyme and lactoferrin, suggesting that ECIG aerosol, like traditional smoke, diminishes the antimicrobial potential of saliva. Another study (Stewart et al., 2018) demonstrated that aerosolized E-liquid could possibly alter oral microbial populations. A recent study demonstrates a significant shift in the beta-diversity of the oral microbiota in ECIG users (Pushalkar et al., 2020). Previous studies from our group have explored the effects of flavorless ECIG aerosol with and without nicotine, and reported that ECIG aerosols have a less detrimental effect on the survival and growth of oral commensal streptococci than conventional cigarette smoke (Cuadra et al., 2019; Nelson et al., 2019), albeit the effects of flavorings were not explored.

In the current study, we evaluate the effects of various commercially available E-liquid flavorings on the growth of the four aforementioned early commensal bacterial colonizers. The aim of this investigation is to test for the effects of common E-liquid flavorings, in a concentration range typically vaped, on the planktonic growth of oral commensal streptococci. We hypothesize that E-liquid flavorings have the potential to alter growth patterns of common commensal oral streptococci. Based on the results of this exploratory investigation, more sensitive and advanced techniques, such as the use of open systems or analysis of three-dimensional oral biofilm scaffolding, will be employed to pin-point specific effects flavoring agents have on polymicrobial communities within the oral cavity. Determining the potential harmful effects of flavoring agents on the growth of oral commensal bacteria is critical to understanding the overall impact of ECIG use on oral health. Oral health is intrinsically tied to systemic health, and maintaining a healthy oral cavity is dependent on the well-balanced growth of the oral microbiota.

## Materials and Methods

### Reagents and Supplies

All reagents and supplies were purchased from Thermo Fisher Scientific (Waltham, MA, United States) unless otherwise noted.

### Bacterial Strains

*Streptococcus gordonii* DL1, *Streptococcus mitis* UF2, *Streptococcus intermedius* 0809 and *Streptococcus oralis* SK139 were generously donated by Dr. Robert Burne from the University of Florida, College of Dentistry in Gainesville, FL, United States. All strains were grown in brain heart infusion (BHI) media and supplemented with 5 μg/mL of bovine hemin or on BHI agar at 37°C and 5% CO_2_ (Rogers and Scannapieco, 2001; Tomoyasu et al., 2010; Huang et al., 2018; Harth-Chu et al., 2019; Hanel et al., 2020). Bacteria stocks were stored at −80°C and purity was validated by Gram stains and light microscopy.

### Stock E-Liquid

**FIGURE 1:**
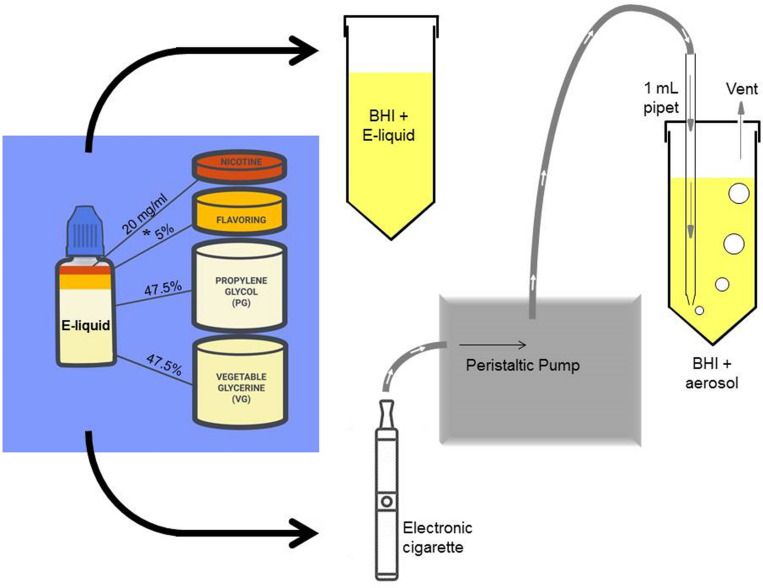
A simple schematic of the experimental procedure depicting the addition of laboratory-prepared stock E-liquid (propylene glycol and vegetable glycerine) containing 5% flavorings (low concentrations of tobacco, menthol, cinnamon, strawberry or blueberry) and 20 mg/ml nicotine into BHI growth media. The * indicates a second stock E-liquid prepared with 25% flavorings (high concentration). The stock E-liquids are introduced into the BHI directly (1, 1.25, 2.5, or 5% E-liquid to BHI ratio) or pumped into the BHI as an ECIG-generated aerosol. One hundred 5-sec puffs of the E-liquid represents about 1% E-liquid in BHI. A portion of this figure is adapted from Nelson et al. (2019).

In Figure 1, stock solutions of E-liquid were prepared using propylene glycol and vegetable glycerin (aka glycerol) in a 1:1 v/v ratio. Concentrated tobacco, menthol, cinnamon, strawberry and blueberry E-Liquid flavors, reconstituted in propylene glycol, were obtained from Liquid Nicotine Wholesalers (Phoenix, AZ, United States) and are described in Table 1. For this investigation, tobacco and menthol flavors were chosen because they simulate conventional cigarette use. According to local vape shop merchants and college students, cinnamon, strawberry, blueberry and other fruity flavors are popular among young adult ECIG users and is the reason they were also chosen for this study. Furthermore, the CDC (Wang et al., 2020) confirms these fruity preferences among youths. As shown in Figure 1, flavored and unflavored E-Liquids were all spiked with 20 mg/mL (S)-(-)-nicotine (Alpha Aesar, Tewksbury, MA, United States). As shown in Table 2, flavored stock E-liquids were prepared as 5% (low concentration) and 25% (high concentration) solutions.

**TABLE 1:** Description of commercially purchased concentrated E-liquid flavoring.

Concentrated flavor	Date purchased	Lot Number	Production or expiration date	Primary diluent	Other ingredients	Comparative coloring	Absorbance reading at 595 nm^∧^
Tobacco	3/30/2019 8/23/2019	L44929# L44929*	NA NA	Propylene Glycol	Natural flavoring, water	Clear	0.043# 0.038*
Menthol	3/30/2019 8/23/2019	192301# 192006*	NA NA	Propylene Glycol	Natural and artificial flavoring	Clear	0.043# 0.039*
Cinnamon	3/30/2019 8/23/2019	CA192005# 93369283*	NA NA	Propylene Glycol	Natural and artificial flavoring, water	Clear Amber	0.152# 0.086*
Strawberry	3/30/2019 8/23/2019	190201# 190905*	NA NA	Propylene Glycol	Natural and artificial flavoring	Clear	0.043# 0.039*
Blueberry	3/30/2019 8/23/2019	181812# 190104*	NA NA	Propylene Glycol	Natural and artificial flavoring	Clear	0.044# 0.039*

# = used in Cuadra Lab; * = used in Palazzolo Lab; NA = not available.∧ = absorbance reading taken of 200 μL of concentrated flavoring in a 96 well plate.

**TABLE 2:** Percentages of Stock E-liquids ± Flavorings in BHI.

Stock E-liquids	Constituents in stock E-liquids				Percent flavoring in BHI after the addition			
		
					of 5, 2.5, 1.25 and 1% of Stock E-liquids			
	Propylene Glycol	Vegetable Glycerine	Flavoring	Nicotine	5%	2.5%	1.25%	1%
No flavoring	50%	50%	0%	20 mg/mL	0%	0%	0%	0%
Low concentration flavoring	47.5%	47.5%	5%	20 mg/mL	0.25%	0.125%	0.0625%	0.05%
High concentration flavoring	37.5%	37.5%	25%	20 mg/mL	1.25%	0.625%	0.3125%	NU

*NU = not used.*

### Kirby Bauer Assays

As an exploratory avenue, Kirby Bauer assays (Bauer et al., 1959, 1966) were used to probe if concentrated flavoring agents had an effect on bacterial growth patterns. Bacteria were grown overnight in BHI media to optical density (OD) of 1.0 reading at 595 nm wavelength. Using sterile cotton swabs, BHI agar plates were inoculated using pure cultures, generating a confluent lawn. Six-millimeter paper disks (BD, Franklin Lakes, NJ, United States) were placed on confluent lawns (*n* = 3 disks per treatment group). Ten microliters of either concentrated flavorings (100%) or stock E-liquid with 5% concentrated flavorings were pipetted onto each disk and allowed to diffuse onto the cultures. Ten microliters of hydrogen peroxide or flavorless stock E-liquid were used as controls. Agar plates were incubated at 37°C and 5% CO_2_ overnight for bacterial growth. The next day, zones of inhibition (ZOI) were visually inspected, and their diameters were measured in millimeters.

### Growth Curves

Two growth curve experiments were conducted. In the first experiment, the effect of 100 puffs of ECIG-generated aerosol were compared to the effect of 1% stock E-liquid ± low concentration (0.05% final percentages in BHI) flavorings, while the second experiment tested for dose responses using stock flavorless E-liquid or E-liquids with low concentration or high concentration flavorings Table 2. As shown in Figure 1, fresh, sterile BHI media (10 ml in 50 ml plastic conical tubes) were supplemented with 1, 1.25, 2.5, or 5% E-Liquid ± low or high concentration flavorings and stored overnight in the refrigerator (4°C), following the methodology of Nelson et al. (2019), which reports no profound differences in the overall growth kinetics of three of the four species tested. Moreover, in order to make our experiments more physiologically relevant, the percentages of stock E-liquid ± flavorings chosen were based on calculations determined from a hypothetical open-system model as outlined in Table 3. According to a previous study (Palazzolo et al., 2017), 9.3 μL of E-liquid is vaporized per puff and there are four puffs per minute (see section “Aerosol Trapping” below). Son et al. (2020), determined that the deposition fraction of ECIG aerosol in the tracheobronchial and bronchoalveolar regions were 0.504-0.541 and 0.073-0.306, respectively, leaving less than 0.400 to be deposited in the oral cavity (Son et al., 2020). From “Saliva and Oral Health, fourth Edition” (Smith, 2012), salivary flow rates range from 0.310 to 0.390 mL/minute. Consequently, the percentage of E-liquid in saliva in this hypothetical open system model (with continuous salivary flow) ranges from 3.5 to 4.3%, which falls within the range of percentages of stock E-liquid used in this study. Consequently, 100 μL (i.e., 1%) of E-liquid ± low concentration flavorings was added directly to the BHI and stored overnight in the refrigerator. As a comparison, one hundred 5-second puffs of stock E-liquid ± low concentration flavorings were bubbled into the BHI media (see section “Aerosol Trapping” below) and also stored overnight at 4°C. Five percent flavorless E-liquid in BHI and 5% hydrogen peroxide in BHI served as the controls. Additionally, 100 puffs of air served as a control for the ECIG-generated aerosol experiment. The following morning, overnight bacterial starter cultures were adjusted to an OD 595 nm of 1.0 by diluting with fresh, sterile BHI media. A final inoculum of 100 μL of adjusted bacterial cultures was added to 10 mL of refrigerated BHI media (1% v/v). In the second experiment, all experimental conditions were identical to the first experiment except that dose-response growth curves were generated using only E-liquid ± low or high concentrations of flavorings added directly to the BHI (i.e., no ECIG-generated aerosol). Three hundred microliters of each inoculated sample, *n* = 12 for the aerosol vs. E-liquid experiment and *n* = 4 to 8 for the dose-response experiments, along with their respective controls, were deposited in 96-well round bottom plates or 96-well flat bottom plates, respectively. For the aerosol vs. E-liquid growth curves, absorbance readings at OD 595 nm were measured at 0, 2, 4, 6, 8, and 24 h using a Thermo Scientific Evolution 300 Ultra Violet-Visible Spectrophotometer (Waltham, MA) with VISIONpro^TM^ software (Conex, Natick, MA, United States). For the dose-response growth curves, absorbance readings at OD 595 nm were measured at 0, 2, 4, 6, 8, 10, 12, and 24 h using a μQuant monochromatic microplate reader equipped with KC4 software version 3.4 (MTX Lab Systems, Bradenton, FL, United States). For both experiments, growth curve samples were incubated at 37°C and 5% CO_2_ for the duration of the experiment, except for the short period of time it took to obtain the absorbance readings. While absorbance readings obtained from round bottom 96-well plates tended to be higher than those obtained in flat bottom 96-well plates, the overall trend of the growth curves was similar as shown in Supplementary Figure 1.

**TABLE 3:** E-liquid/saliva in a hypothetical open system and E-liquid/BHI in a closed system.

	Open system		Closed system	
Volumes of flavorless E-liquid and Saliva in a model open system	High range	Low range		
Volume of E-liquid in 1 minute (i.e., 4 puffs)*	37.2 μL	37.2 μL		
Volume of E-liquid in 1 minute deposited into the oral cavity (<40%)^@^	14.9 μL	14.9 μL		
Volume of unstimulated saliva after 1 minute^#^	310 μL	390 μL		
Volume of E-liquid and unstimulated saliva after 1 minute	324.9 μL	404.9 μL		
Percent E-liquid in Saliva of oral cavity	4.6%	3.7%		

**Volumes of flavorless E-liquid and BHI used in this study**			**High range**	**Low range**

Volume of E-liquid			0.5 mL	0.1 mL
Volume of BHI			9.5 mL	9.9 mL
Volume of E-liquid and BHI			10 ml	10 mL
Percent of E-liquid in BHI			5.0%	1.0%

*Palazzolo et al. (2017).^@^Son et al. (2020).^#^Smith (2012).

### Aerosol Trapping

As previously described (Nelson et al., 2019), E-liquid was aerosolized using a Tripl3 (Kennesaw, GA, United States) eGo style lithium ion battery (650 mAh, 3.7 V unregulated). The E-liquid was housed in a 1.8 mL capacity Aspire glass tank (Shenzhen Eigate Technology Co., Ltd., Shenzhen, China) equipped with a 1.8 Ω resistance coil for an average power output of ≈ 7.6 W. Air or ECIG-generated aerosol ± flavorings were delivered into 10 ml of BHI using a Cole-Palmer Master Flex L/S peristaltic pumps (Vernon Hills, IL, United States). Tubing retrofitted onto 1 mL serologic pipettes delivered aerosolized E-liquid directly into BHI media through bored holes into closed but vented 50 mL conical tubes (Figure 1). Flow rate was adjusted to 400 mL/minute (i.e., 33.3 mL per five second puff). Puffing was achieved by activating the pump for five seconds (pump on) followed by a ten second rest period (pump off). The puffing protocol consisted of 100 puff cycles (pump on/off). Using this methodology, 9.3 μL of E-liquid is aerosolized per puff, or 930 μL for 100 puffs (Palazzolo et al., 2017). Since it was determined that the percent recovery of aerosolized E-liquid in the BHI is between 8.4 and 10.1% (Nelson et al., 2019), the amount of aerosolized E-liquid that is present in the BHI ranges between 78 and 94 μL. Consequently, 100 μL of E-liquid added directly to the 10 ml of BHI (or 1%) is roughly equivalent to 100 puffs. All aerosol trapping was conducted within a P20 Purair ductless fume hood (Airscience, Fort Meyers, FL, United States) with a high-efficiency particulate air (HEPA) filter. While we fully recognize that our puffing regimen does not follow guidelines specified by the CORESTA recommended method N°81,^2^ we opted to use our puffing regimen for the sake of comparison and consistency with our previous two publications (Cuadra et al., 2019; Nelson et al., 2019).

### Statistical Analysis

All experimental and control data points in the Kirby Bauer assays and in the bacterial growth curves were analyzed for means and standard error of means (SEM). Additionally, Supplementary Table 1 reports all means and standard deviations for all data points in the Kirby Bauer assays and in the bacterial growth curves. For growth curves comparing the effect of 100 puffs of ECIG-generated aerosol with the effect of 1% stock E-liquid ± low concentration (0.05% final percentages in 10 mL of BHI) flavorings, data points for the exponential phase of growth curves (2–6 h for *S. gordonii* and *S. mitis*, and 4–8 h for *S. intermedius* and *S. oralis*) were subjected to log transformations followed by linear regression analysis. *F*-tests were used to determine differences between regression line slopes comparing E-liquid or ECIG-generated aerosol with vs without flavorings. Statistical differences between treatment groups in the Kirby Bauer assays, growth curve analysis and regression line slope analysis was established using one-way analysis of variance (ANOVA) followed by Tukey’s *post hoc* analysis. A *p* < 0.05 was considered significant. PRISM 5 (GraphPad Software, San Diego, CA, United States) was used to perform all statistical calculations.

## Results

### Kirby Bauer Assays

**FIGURE 2:**
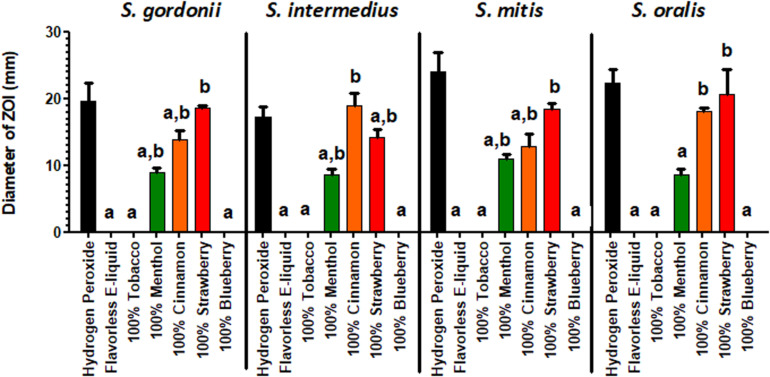
Kirby-Bauer assays depicting the effects of 100% concentrated flavorings in E-liquid on the Zone of Inhibition. Each bar represents mean ± SEM, *n* = 3 is the number of replicates. *a* = *p* < 0.05 from hydrogen peroxide (positive control) and *b* = *p* < 0.05 from negative control (flavorless E-liquid).

The effect of E-liquid flavorings on the growth of commensal streptococci on BHI agar plates is shown in Figure 2. As demonstrated by increased ZOIs, growth of commensal streptococci species on BHI agar was significantly inhibited when exposed to 100% concentrated menthol (*S. oralis* was the exception), cinnamon and strawberry flavors, as compared to the flavorless E-liquid control. Furthermore, in many instances, the 100% concentrated cinnamon (for *S. oralis*) and strawberry (for *S. gordonii*, *S. mitis*, and *S. oralis*) treatments yielded ZOIs comparable to that of the hydrogen peroxide control. In contrast, as shown in Supplementary Figure 2, none of the commensal streptococci species, when exposed to 5% flavorings diluted in stock E-liquid base, exhibited a statistical difference in ZOIs when compared to the flavorless E-liquid control. The data indicate that concentrated flavorings are toxic to oral bacteria. Since E-liquids containing 5% flavorings are more realistic doses to human consumption, the Kirby Bauer methodology is not sensitive enough to test inhibitory effects of E-liquids on the growth of oral commensal bacteria.

### Growth Curves: Comparison of ECIG-Generated Aerosol and E-Liquid on Planktonic Growth of Oral Commensal Bacteria

**FIGURE 3:**
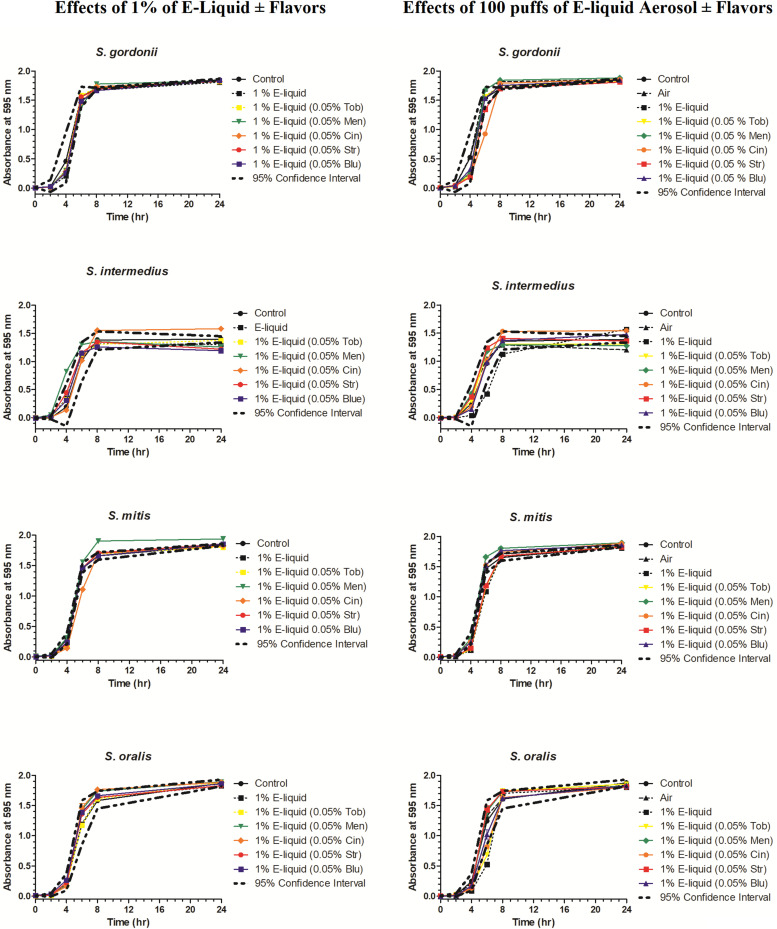
Effects of 100 μL E-liquid ± flavorings (1% in BHI) added directly into BHI (graphs on left) and 100 puffs of ECIG-generated aerosol ± flavorings (approximately 1%) pumped into BHI (graphs on right) on streptococcal 24-h growth curves. Each point represents Mean ± SEM, *n* = 12 is the number of replicates.

**FIGURE 4:**
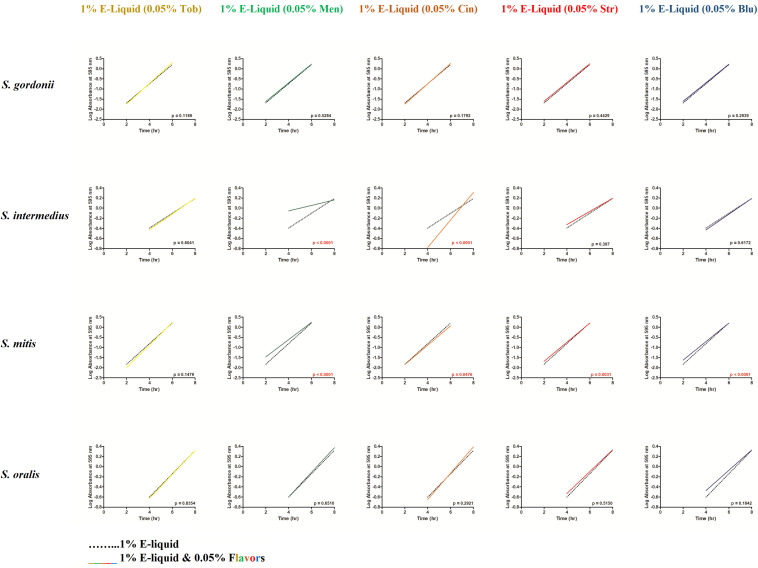
From the Figure 3 data, linear regressions were determined depicting the effects of 100 μL of E-liquid ± flavorings on bacterial growth during the log phase of the 24-h growth curves (i.e., between 2 and 6 h for *S. gordonii* and *S. mitis* and between 4 and 8 h for *S. intermedius* and *S. oralis*). One hundred microliters of E-liquid in 10 mL of BHI = 1%. The flavoring present in the E-liquid = 0.05%. Within each graph panel (i.e., bacteria/flavoring combination), red *p*-values (*p* < 0.05) indicate the slopes of the regression lines are significantly different. Each slope is calculated from 36 data points (3 time points X 12 replicates for each time point).

**FIGURE 5:**
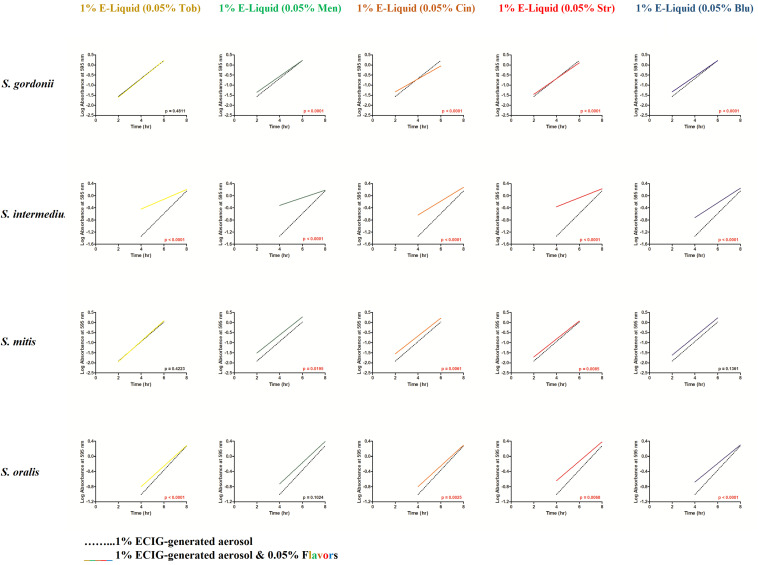
From the Figure 3 data, linear regressions were determined depicting the effects of 100 puffs of ECIG-generated aerosol ± flavorings on bacterial growth during the log phase of the 24-h growth curves (i.e., between 2 and 6 h for *S. gordonii* and *S. mitis* and between 4 and 8 h for *S. intermedius* and *S. oralis*). One hundred puffs of ECIG-generated aerosol in BHI ≈ 1%. The flavoring present in the ECIG-generated aerosol = 0.05%. Within each graph panel (i.e., bacteria/flavoring combination), red *p*-values (*p* < 0.05) indicate the slopes of the regression lines are significantly different. Each slope is calculated from 36 data points (3 time points X 12 replicates for each time point).

To gain more insight into the effects of E-liquid flavorings, we conducted planktonic growth curves, first comparing E-liquid pipetted directly into BHI vs. ECIG-generated aerosol bubbled into the media as illustrated in Figure 1. The left-hand graphs of Figure 3 show1% concentration of stock E-liquid ± flavorings in BHI, which corresponds to 0.05% flavoring concentration (Table 3), for all bacterial 24-h growth curves. The results show that all conditions tested yielded growth patterns similar to untreated controls. Likewise, the right-hand graphs of Figure 3 illustrate that 100 puffs (approximation of 1% stock E-liquid) of ECIG-generated aerosol ± flavorings for all bacterial 24-h growth curves were similar to both 100 puffs of air and untreated controls. Furthermore, most of the points for all treatment curves fell within the 95% confidence interval of the control curves (*n* = 12) and one-way ANOVA indicates no statistical differences between any of the curves. In order to further evaluate growth rates during exponential phase, linear regression analyses of this interval for each bacteria/flavoring combination are shown in Figure 4 (1% E-liquid ± flavorings) and Figure 5 (100 puffs of E-CIG generated aerosol ± flavorings). In Figure 4, the linear regression lines for *S. intermedius* exposed to menthol and cinnamon have slopes that are statistically different from flavorless E-liquid. Similarly, the regression lines for *S. mitis* exposed to menthol, cinnamon, strawberry and blueberry have slopes that are statistically different from flavorless E-liquid. In Figure 5, the linear regression lines for *S. gordonii* exposed to menthol, cinnamon, strawberry and blueberry have slopes that are statistically different from flavorless E-liquid. The regression lines for *S. intermedius* exposed to tobacco, menthol and cinnamon, and the regression lines for *S. mitis* exposed to menthol, cinnamon and strawberry have slopes that are statistically different from flavorless E-liquid. Finally, regression lines for *S. oralis* exposed to tobacco, cinnamon, strawberry and blueberry have slopes that are statistically different from flavorless E-liquid. Table 4 summarizes the effects of 1% of flavored E-liquid (Figure 4) and 100 puffs of flavored ECIG-generated aerosol (Figure 5) on all four bacteria tested. Slightly more than half of the comparisons between flavored and unflavored treatments revealed significance. Of those significant comparisons, all but one indicated inhibition of growth (i.e., shallower slope). Furthermore, the flavored ECIG-generated aerosol resulted in 15 significant slope differences, while the flavored E-liquid only resulted in six significant slope differences. From these results, it appears that bacteria exposed to the aerosol grow slower during the exponential phase than bacteria exposed to the unaerosolized E-liquid. When the slopes generated in Figures 4, 5 were pooled, either by bacterial species (*n* = 10) or by flavoring (*n* = 8), no statistical differences in the slopes were detected between groups (Supplementary Figure 3). Even though all species reach stationary phase under all treatments, these results indicate the possibility that flavorings, in general, may slow the growth of the bacteria during the exponential phase. Strikingly, ECIG-generated aerosol seems to hinder the growth of the four species tested. Overall, our data indicate that flavored aerosols from ECIGs seem to affect the growth of oral commensal bacteria.

**TABLE 4:** Effect of flavorings on bacterial growth based on combined linear regression analysis obtained from combined Figure 4 (exposure to E-liquid directly) and Figure 5 (exposure to ECIG-generated aerosol) data.

	Tobacco Figures 4, 5	Menthol Figures 4, 5	Cinnamon Figures 4, 5	Strawberry Figures 4, 5	Blueberry Figures 4, 5	Number of slopes where *p* < 0.05
*S. gordonii*	0 & 0	0 & −1	0 & −1	0 & −1	0 & −1	4
*S. intermedius*	0 & −1	−1 & −1	+1 & −1	0 & −1	0 & −1	7
*S. mitis*	0 & 0	−1 & −1	−1 & −1	−1 & −1	−1 & 0	7
*S. oralis*	0 & −1	0 & 0	0 & −1	0 & −1	0 & −1	4
Number of slopes where *p* < 0.05	2	5	6	5	4	22 of 40 slopes have *p* < 0.05

*0 = slopes are not significantly different.1 = slopes are significantly different (p < 0.05).− = inhibited growth (flavoring slope is shallower).+ = stimulated growth (flavoring slope is steeper).*

### Growth Curves: Dose-Dependent Effect of Flavored Stock E-Liquids

**FIGURE 6:**
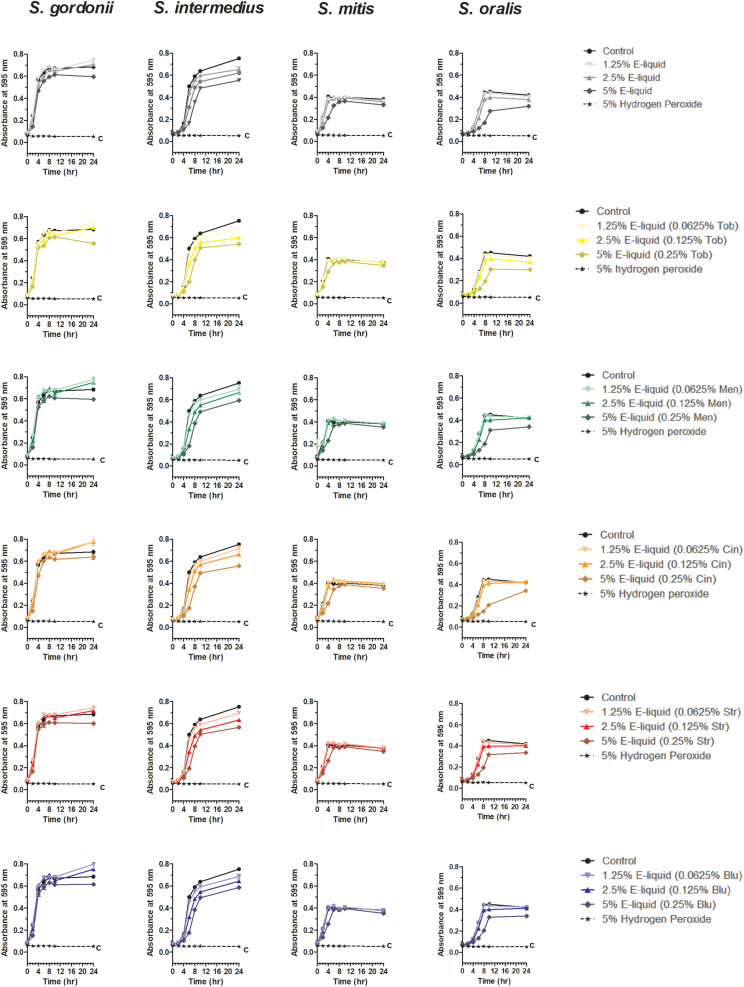
Twenty-four hour growth curves illustrating dose responses of E-liquid ± low concentration flavorings. Each point represents mean ± SEM, *n* = 4 to 8 is the number of replicates. *c* = *p* < 0.001 from untreated control.

**FIGURE 7:**
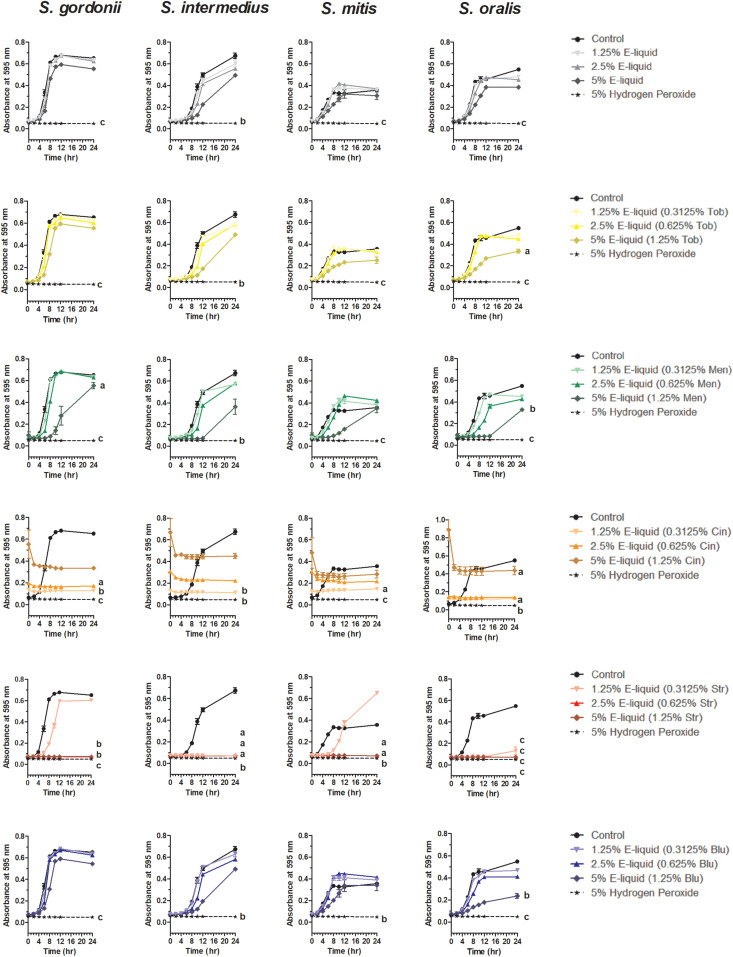
Twenty-four hour growth curves illustrating dose responses of E-liquid ± high concentration flavorings. Each point represents mean ± SEM, *n* = 4 is the number of replicates. *a* = *p* < 0.05, *b* = *p* < 0.01, and *c* = *p* < 0.001 from untreated control. For a complete list of comparative statistics see Supplementary Table 2.

**FIGURE 8:**
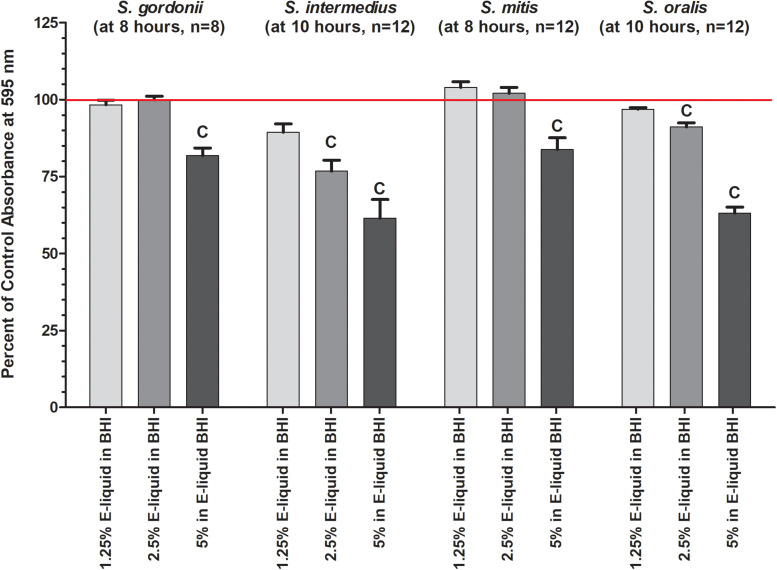
Effects of percent flavorless E-liquid in BHI on bacterial growth at the start of the plateau phase. Each bar represents mean ± SEM percent change from control where n, as shown in the graph, is the number of replicates. Red line indicates 0% E-liquid (control). *c* = *p* < 0.001 from 0 % E-liquid.

**FIGURE 9:**
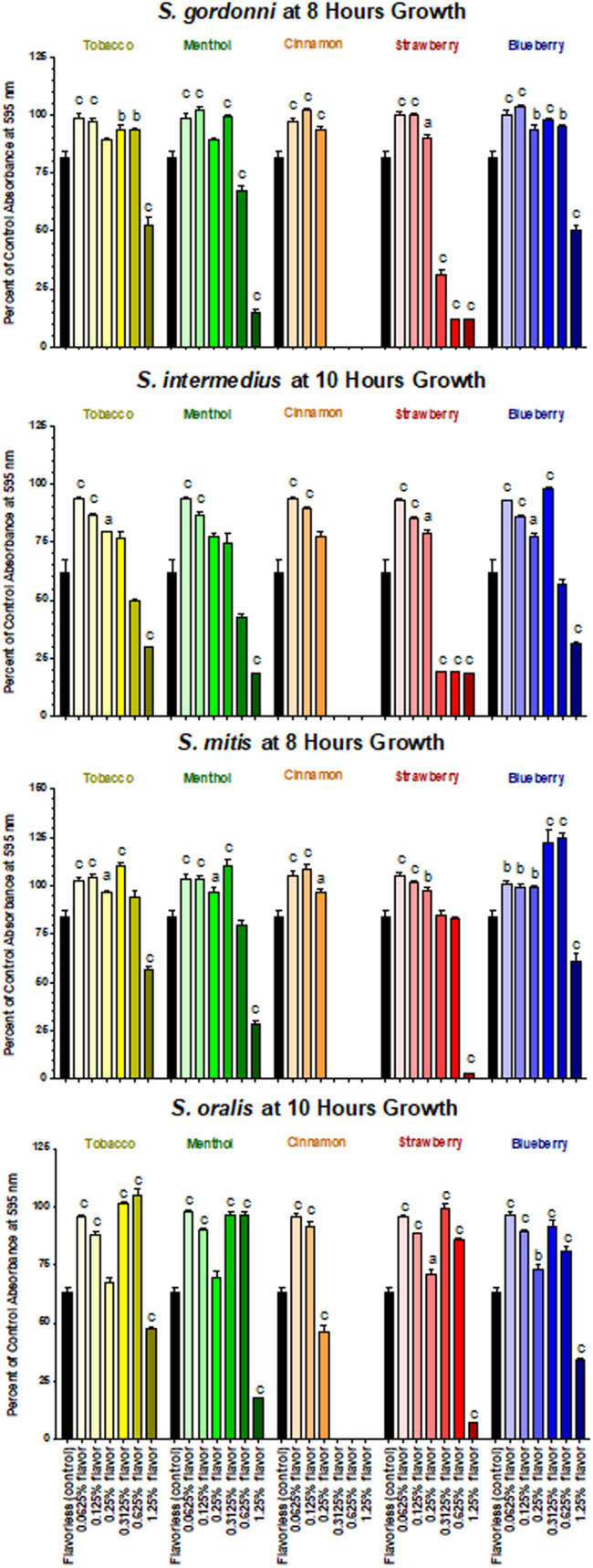
Effects of E-liquid ± high and low percent flavorings in BHI on planktonic bacterial growth. Each bar represents Mean ± SEM percent change from the planktonic growth in BHI control media (no E-liquids), *n* = 4 to 12 are the number of replicates. *a* = *p* < 0.05 from flavorless E-liquid, *b* = *p* < 0.01 from flavorless E-liquid and *c* = *p* < 0.001 from flavorless E-liquid.

Based on the results of the Kirby Bauer assays, where 100% of the menthol, cinnamon and strawberry flavors inhibited bacterial growth while 5% flavorings in E-liquid had no effect; dose-response experiments were conducted to determine the percentage of flavoring in E-liquid required to inhibit planktonic bacterial growth. Figure 6 illustrates the effects of low concentration (0.0625, 0.125, and 0.25%) flavoring on the growth of four strains of oral commensal bacteria. While none of the bacteria/flavoring combinations exhibited statistical significance from the control growth curves, there was a clear tendency for higher flavoring doses to delay growth. In contrast, Figure 7 demonstrates that high concentration (0.3125, 0.625, and 1.25%) flavoring exert statistically significant dose-dependent effects, especially for menthol, cinnamon and strawberry. On initial interpretation, it appears that cinnamon has a reverse dose effect, but this is not the case. Since concentrated cinnamon has a higher absorbance reading (i.e., is darker) than the other flavorings (see Table 1), addition of 25% concentrated cinnamon flavor to the E-liquid inherently increases the initial absorbance readings of the growth media, thus giving the appearance of a reverse dose effect. In actuality, the high concentrations (0.3125, 0.625 and 1.25%) of cinnamon completely impair bacterial growth. A complete list of comparative statistics for Figure 7 is outlined in Supplementary Table 2. Based on early stationary phase for each streptococci (8 h for *S. gordonii* and *S. mitis* or 10 h for *S. intermedius* and *S. oralis*), comparisons of all absorbance values are shown in Figure 8 as a percent of the corresponding control values (i.e. no E-liquid). Increasing the percentage of flavorless E-liquid in BHI from 1.25 to 5% significantly (*p* < 0.001) inhibits the growth of all bacteria tested. Figure 9 illustrates the effects of flavored E-liquids by early stationary phase for each streptococci (0.0625, 0.125, 0.25, 0.3125, 0.625, and 1.25 final percentages in BHI) as compared to 5% flavorless E-liquid in BHI. For all streptococci, the lowest percent of all flavorings in BHI exhibited statistically higher values than 5% flavorless E-liquid, while the highest percent of all flavorings in BHI exhibited statistically lower values than 5% flavorless E-liquid. Since concentrated cinnamon has a higher absorbance reading than the other flavorings (Table 1), absorbance values for the high percentage cinnamon flavored E-liquid (0.3125, 0.625, and 1.25 final percentage in BHI) were higher than control values (no E-liquid) and were subsequently normalized to control baseline. When expressed as a percent of control, all values, except one (0.3125% cinnamon for *S. mitis*), were negative (Supplementary Figure 4) and consequently zero values are reported in Figure 9. In summary, these results indicate that flavorless E-liquid at concentrations higher than 2.5% in BHI decrease bacterial growth. In addition, low concentrations of flavored E-liquids appear to increase bacterial growth while high concentrations of flavored E-liquids decrease bacterial growth. Altogether, our data suggest that E-liquids and their aerosols ± flavorings alter the growth patterns of oral commensal bacteria *in vitro*. Such growth alterations have the potential to ultimately affect balance of multi-species oral biofilms and could lead to dysbiosis and disease.

## Discussion

This work expands upon our previous discoveries and introduces, for the first time, the effects of flavoring compounds on the growth of oral commensal bacteria by assaying species independently in solid state growth on BHI agar and in BHI liquid cultures. For these studies, concentrations of all flavorings ranged between 5 and 25% of the total E-liquid solution (Table 2), typical for most ECIG users. Additionally, the percentage of E-liquid in BHI ranged between 1 and 5%, a close approximation to the percentage of E-liquid (as aerosol) one might find in saliva lining the oral cavity (Table 3). Under these conditions, flavoring agents were shown to have an inhibitory effect on the growth of all four oral species tested. The data reported here not only agree with our previous findings on the negligible effects of 1% flavorless E-liquid on oral commensals (Cuadra et al., 2019; Nelson et al., 2019), but also focus on the potential dangers of higher concentration E-Liquids ± flavorings and their aerosols on the growth of oral streptococci. Full strength flavorings, but not 5% flavorings in E-liquid, were observed to have an inhibitory effect on Kirby Bauer assays, highlighting this technique’s lack of sensitivity (Figure 2 and Supplementary Figure 2). Among the tested flavors, menthol, cinnamon and strawberry were observed to have significant inhibitory effects on the growth of oral species on BHI agar. Although 24-h planktonic growth curves for all bacteria/flavoring combinations were similar (Figure 3), regression analyses of the exponential growth intervals were disparate when treated with both E-liquid ± flavorings (Figure 4) and ECIG-generated aerosol ± flavorings (Figure 5). Low concentration flavorings in E-liquid were observed to have a dose-dependent, yet not statistically significant effect (Figure 6). On the other hand, high concentration flavorings in E-liquid cause a dose-dependent, and statistically significant, decrease in bacterial growth (Figure 7). Further analysis of these data at late exponential phase demonstrate that concentrations higher than 1% flavorless E-liquid in BHI can contribute to delayed growth of oral commensal bacteria (Figure 8). Similarly, E-liquid flavorings were observed to have a significant inhibitory effect for all four commensal species across all flavored conditions at high concentrations, particularly menthol, cinnamon and strawberry (Figure 9). This suggests that a homemade 25% flavored E-liquid solution (v/v) used in the course of a day may severely alter the growth of oral bacteria *in vivo*. In terms of real-world vaping, exposure to high concentration flavored E-liquid solutions on the growth of these oral commensal streptococci may depend, not only of the aerosolized E-liquid constituents, but also on user puff topography (Beauval et al., 2019) known to alter the production and emission of various carbonyl compounds, which in turn, could have an effect on commensal bacterial growth. The present study was limited to a 1:1 propylene glycol to glycerol ratio, a nicotine concentration of 20 mg/mL and a single predefined puff topography as specified in the aerosol trapping section of the Materials and Methods. However, previous work from this lab (Nelson et al., 2019) reported that varying the ratio of propylene glycol/glycerol or varying the concentration of nicotine in a flavorless E-liquid did not significantly alter the growth patterns of *S. gordonii*, *S. mitis* and *S. oralis*. Alternatively, an argument can be made that varying humectant ratio or nicotine concentration could either attenuate or amplify the effect E-liquid flavorings have on the growth of these bacterial species. Ultimately, these data demonstrate that E-liquid ± flavorings have a variable and potentially harmful effect on the growth of oral commensal streptococci.

E-liquid compounds, when heated, may contribute harmful byproducts (Lerner et al., 2015; Bitzer et al., 2018; Qu et al., 2018; Strongin, 2019) to the aerosol. Additionally, using ECIGs may lead to the leaching of toxic metals from the heating coil and other metal components of the ECIG device into the aerosol (Kosmider et al., 2014; Lerner et al., 2015; Palazzolo et al., 2017; Bitzer et al., 2018; Olmedo et al., 2018). Furthermore, metals have been reported as toxins to oral streptococci (Dunning et al., 1998). Since bacteria exposed to low concentrations of E-liquids ± flavorings and their respective aerosols have similar growth patterns, despite the fact that growth profiles during the exponential phase appear to exhibit a slight hindrance in growth, these harmful byproducts do not appear to interfere with overall growth, especially at low level exposure. Consequently, our data demonstrate that the dose-dependent E-liquid toxicity is due solely to the E-liquid constituents themselves and not to trace metals or other byproducts leached from the ECIG device. Any amount of inhibition resulting from aerosolized byproducts and metals liberated from the ECIG device is consistent across all experimental groups and therefore cannot be implicated for growth inhibition in this study. However, this does not preclude the possibility that these byproducts may affect transcriptional regulation or enzymatic activity. For example, transcriptomic analysis of genes such as *recA* and *lytA* (Lewis, 2000), which respond to DNA from lysed cells, as well as stress genes such as *sdbA* (Davey et al., 2016), may reveal further understanding of the adverse effects of E-liquid flavorings on commensal streptococci.

To date, there are few studies dealing with the effects of E-liquid flavorings on the oral microbiota. One study analyzed the effects of E-liquids on *S. mutans* and found accelerated growth on this cariogenic species as high-sucrose, gelatinous candies and acidic drinks (Kim et al., 2018). Another study examined the oral and gut microbiota of 30 humans and found no significant beta diversity between ECIG users and the control group (Stewart et al., 2018). Of clinical relevance to the oral cavity among ECIG users are recent reports demonstrating the presence of oral mucosal lesions, lacerations, and dental avulsions (GülŞen and Uslu, 2020), as well as nicotine stomatitis (commonly known as smoker’s palate), a hairy tongue and inflammation of the lips, a condition known as angular cheilitis (Bardellini et al., 2018). Strikingly, measurements of cotinine, the main metabolite of nicotine, in the saliva and urine of second-hand vapers has also been shown to be significantly increased (Ballbè et al., 2014). However, the role of flavors in aerosolized E-liquid on these clinical conditions have yet to be determined. Alternatively, these E-liquid effects have been characterized on a variety of mammalian tissues and cell lines. E-liquid aerosols containing classic tobacco flavors were found to be potent stimulators of interleukin (IL)-6 and IL-8 in human airway epithelial cells H292 (Lerner et al., 2015). Similarly, human lung fibroblasts displayed stress, morphological changes and high production of IL-8 when treated with E-liquids and aerosols with cinnamon flavors (Lerner et al., 2015). Moreover, murine lung epithelia *in vivo* presented diminished levels of both glutathione (GSH) and glutathione disulfide (GSSG) compared to control, demonstrating impairment of cells, and likely microbes, to maintain proper total glutathione balance (Lerner et al., 2015). This impairment in redox balance could be a potential mechanism through which E-liquids ± flavorings affect microbial growth. In another study (Leigh et al., 2016), many ECIG flavors, including tobacco, menthol and strawberry were found to significantly diminish H292 bronchial epithelial cell viability and metabolic activity when grown *in vitro* (Leigh et al., 2016). Key cytokines such as IL-1β, IL-10 and chemokines, including CXCL1, CXCL2 and CXCL10, were upregulated by strawberry flavor (Leigh et al., 2016). Cinnamaldehyde, the major component of many cinnamon flavors, was shown to decrease viability of human monocytic U937 and MM6 cells and caused upregulation of IL-8 in a dose-dependent manner (Muthumalage et al., 2018). Our data correlate well with the above studies of eukaryotic models in that the effects of E-liquids and their aerosols leading to the aforementioned stress-responses could also be occurring in oral commensal streptococci, and may provide a putative mechanism of growth inhibition.

Many flavorings are food derivatives, but also possess antimicrobial activity. We speculate that ECIG flavorings, as food derivatives, serve as an additional carbon source for bacterial metabolism and growth. Perhaps these putative carbon sources at low concentrations improve oral bacterial growth. Although the exact chemical structure of the flavoring agents are unknown, the probability is high that these molecules are the same as those found in natural botanicals. Oral commensal bacteria are often exposed to these flavoring molecules when humans eat these plants. For example, menthol is found in many terpene-containing herbs (Aggarwal et al., 2015), while cinnamon is frequently used as a cooking enhancement. Strawberries and blueberries are known to contain many beneficial compounds such as antioxidants and vitamins in addition to their natural flavors. Flavoring molecules, which are pleasant to taste at low concentrations could be offensive or even toxic to the human mouth at high concentrations. Consequently, a similar argument could be proposed for the biology of oral commensal bacteria. Oral commensal bacteria exposed to E-liquids with high concentration of flavoring agents (25%) experience diminished growth under conditions similar to those commonly seen with antibiotics. For example menthol and cinnamaldehyde are known to be toxic to bacteria and are avowed anti-microbials (Solórzano-Santos and Miranda-Novales, 2012; Freires et al., 2015). Importantly, oral commensal bacteria have developed significant multidrug resistance, based on the long-term usage of antibiotics in medicine (Thornton et al., 2015). The development of multidrug resistance by commensal streptococci species suggests the potential for these commensals to develop resistance to E-liquid aerosols containing high concentrations of flavorings. As a matter of speculation, these results suggest that when exposed to low concentrations of flavoring agents, oral bacteria either adapt and possibly over-compensate their growth or use these compounds as an additional source of nutrients. In either case, low concentrations of flavoring agents induce faster growth rates. Alternatively, high concentrations appear to act as antimicrobials reducing growth rates of these oral commensals. Ultimately, low-level exposure to flavored ECIG aerosol may induce faster oral commensal growth *in situ*, which in itself is a disruption of the oral microbial ecology, while high-level exposure of flavoring agents decrease the growth of oral commensal bacteria. Regardless of high- or low-level exposure, flavored-induced alterations in bacterial growth in the oral microbial environment could lead to changes in host-bacteria interactions and may contribute to dysbiosis, thus promoting the onset of oral disease.

The present study was limited to investigation of four oral commensal species that inhabit the human oral microbiome. These commensal streptococci were studied because they strongly represent the biomass of the beginning stages of oral biofilm formation, when accounting for the raw percentage of these four species (Colombo et al., 2007). Our *in vitro* study attempts to mimic microbial growth in BHI agar and planktonic growth in BHI media exposing bacteria to physiologically relevant concentrations of E-liquids in a closed system. Other studies have shown elegant open systems producing oral biofilms, reflecting a better approximation of microbial growth *in vivo* (Kolenbrander et al., 2006; Rickard et al., 2008; Cuadra-Saenz et al., 2012). While saliva would be the preferred medium, BHI broth has been well validated to support the growth of commensal streptococci and has become commonplace as the standard medium for *in vitro* assays (Kreth et al., 2008; Cuadra et al., 2019; Nelson et al., 2019; Hanel et al., 2020). Additionally, these studies were performed as single-species cultures which takes away the interspecies interactions present in the oral cavity (Socransky et al., 1998; Kolenbrander, 2000; Kolenbrander et al., 2006; Cuadra-Saenz et al., 2012; Diaz and Valm, 2019). More realistic conditions that would allow us to study the effects of flavored E-liquid exposure on oral commensal bacteria would include an open system, growing multi-species biofilms fed solely with a continuous flow of saliva. Moreover, future studies should incorporate the presence of competitive pathogens, mimicking more realistically the oral microbial environment. In our future experiments, our group will explore growth of periodontal bacterial pathogens such as *Fusubacterium nucleatum*, *Porphyromonas gingivalis, Aggregatibacter actinomycetemcomitans* and tongue candidiasis yeast pathogen *Candida albicans* in combination with commensal streptococci under the same experimental conditions. Such studies would help address relevant effects on the interactions between commensal streptococci and oral pathogens. In addition, E-liquid flavorings and their components will be tested for bactericidal, bacteriolytic or bacteriostatic properties on oral bacteria. Molecular studies with low levels of E-liquid exposure will also be explored to identify putative genes involved in metabolism or stress response to these agents. Another limitation is the proprietary nature of the commercial E-liquid flavors themselves, which in turn limit the understanding of flavoring-induced inhibitory mechanisms. Chemical separation and identification of flavoring components via comprehensive HPLC and LCMS/GCMS analysis would be necessary to begin to determine any potential mechanisms of growth inhibition. Future chemical analyses would identify individual compounds within E-liquid flavorings to be tested for microbial inhibition and toxicity.

In conclusion, this study indicates that flavored E-liquid, particularly with higher concentration of flavoring agents, has a significant inhibitory effect on the planktonic growth of oral commensal streptococci at physiologically relevant concentrations and exposures. Our study (at least under conditions of low-level exposure to flavorings) also validates that non-aerosolized E-Liquid serves as a comparable model to its aerosol counterpart. Furthermore, this study paves the way for future studies to continue investigating the effects of flavored ECIG-generated aerosols and E-liquids on oral bacteria and biofilms. Destabilization of the oral microbiota has been implicated in severe disease such as gingivitis, caries, and periodontal disease (Rosan and Lamont, 2000; Kreth et al., 2008; Gross et al., 2012; Marsh et al., 2015). The commensal oral microbiota, specifically *S. gordonii* and *S. intermedius* have been demonstrated to restrict *Porphyromonas gingivalis* invasion into oral epithelia, which may serve as a protective measure against gingivitis (Hanel et al., 2020). Oral disease serves as both a contributor to and predictor of poor systemic health that disseminates beyond the oral cavity and can have lifelong impact on human health and physiology.

## Data Availability Statement

The original contributions presented in the study are included in the article/Supplementary Material. Further inquiries can be directed to the corresponding author.

## Author Contributions

GC and DP conceived the study, designed the experiments, and provided insight to the project. GC and JF performed Kirby Bauer assays. JF, SS, DL, GC, and DP performed the growth curve experiments. DP analyzed the data. JF, GC, and DP contributed to the writing of the manuscript. All authors contributed to the article and approved the submitted version.

## Conflict of Interest

The authors declare that the research was conducted in the absence of any commercial or financial relationships that could be construed as a potential conflict of interest.

## Footnotes

1 It is important to make a distinction between E-liquid flavors versus flavorings; where the former is the sensation perceived by the ECIG user and the latter refers to the actual compounds that result in the sensation of a flavor. Furthermore, most commercially available flavored E-liquids are proprietary and the actual flavoring agents are not made public.

2 https://www.coresta.org/sites/default/files/technical_documents/main/CRM_81.pdf

## Supplementary Material

Supplementary Figure 1 Comparison of 24-hour growth curves using round bottom vs flat bottom 96-well plates. Each point represents Mean ± SEM, *n* = 12 is the number of replicates.

Supplementary Figure 2 Kirby-Bauer assays depicting the effects of 5% of the concentrated flavorings in E-liquid on the Zone of Inhibition. Each bar represents mean ± SEM, *n* = 3 is the number of replicates. *a* = *p* < 0.05 from hydrogen peroxide (positive control) and *b* = *p* < 0.05 from negative control (flavorless E-liquid).

Supplementary Figure 3 Percent change in slope (combined data from Figures 4, 5) as compared to flavorless E-liquid; by bacterial species (upper panel) and by flavor (lower panel). Each bar represents mean ± SEM, *n* = 8 in upper panel and *n* = 10 in lower panel are the number of replicates.

Supplementary Figure 4 Each bar represents mean ± SEM of the percent of control absorbance (OD 595) readings for all bacteria exposed to high concentration cinnamon flavor where *n*, as shown in the graph, is the number of replicates.

Supplementary Table 1 Means ± SD and *n*-values for all data points in the Kirby Bauer assays and in the bacterial growth curves.

Supplementary Table 2 Complete list of statistics for Figure 7.
