# Supplementary figures and images for: Flavorless vs. Flavored Electronic Cigarette-Generated Aerosol and E-Liquid on the Growth of Common Oral Commensal Streptococci
Source: Front Physiol. 2020 Nov 23;11:585416. doi: 10.3389/fphys.2020.585416 (PMC7732452; doi:10.3389/fphys.2020.585416)

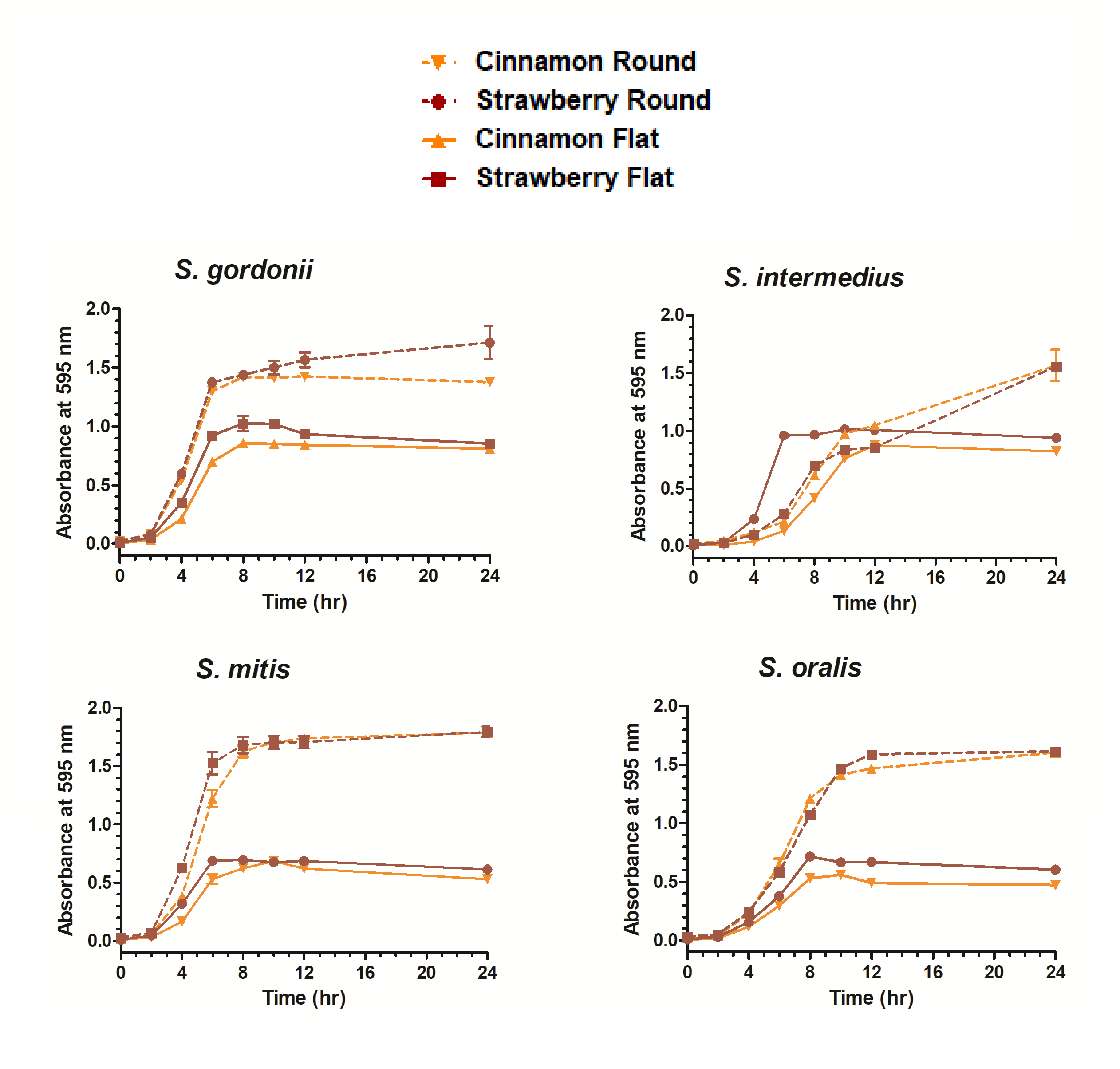

Supplement: Supplementary Figure 1 — Comparison of 24-hour growth curves using round bottom vs flat bottom 96-well plates. Each point represents Mean ± SEM, n = 12 is the number of replicates. [file Image_1.TIF]

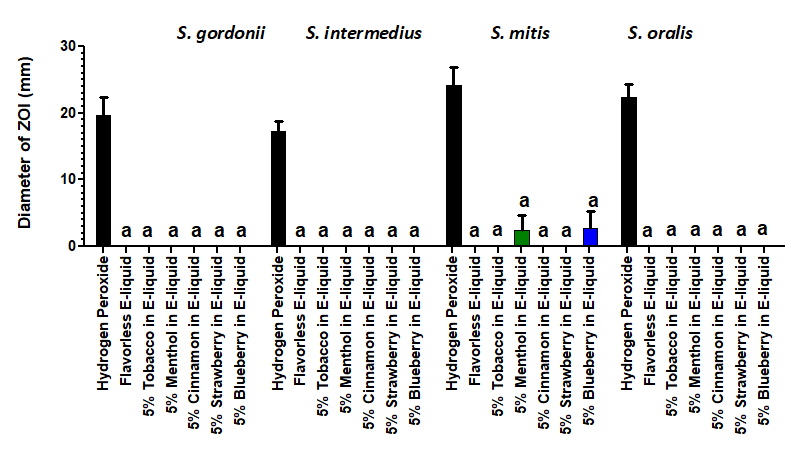

Supplement: Supplementary Figure 2 — Kirby-Bauer assays depicting the effects of 5% of the concentrated flavorings in E-liquid on the Zone of Inhibition. Each bar represents mean ± SEM, n = 3 is the number of replicates. a = p < 0.05 from hydrogen peroxide (positive control) and b = p < 0.05 from negative control (flavorless E-liquid). [file Image_2.TIF]

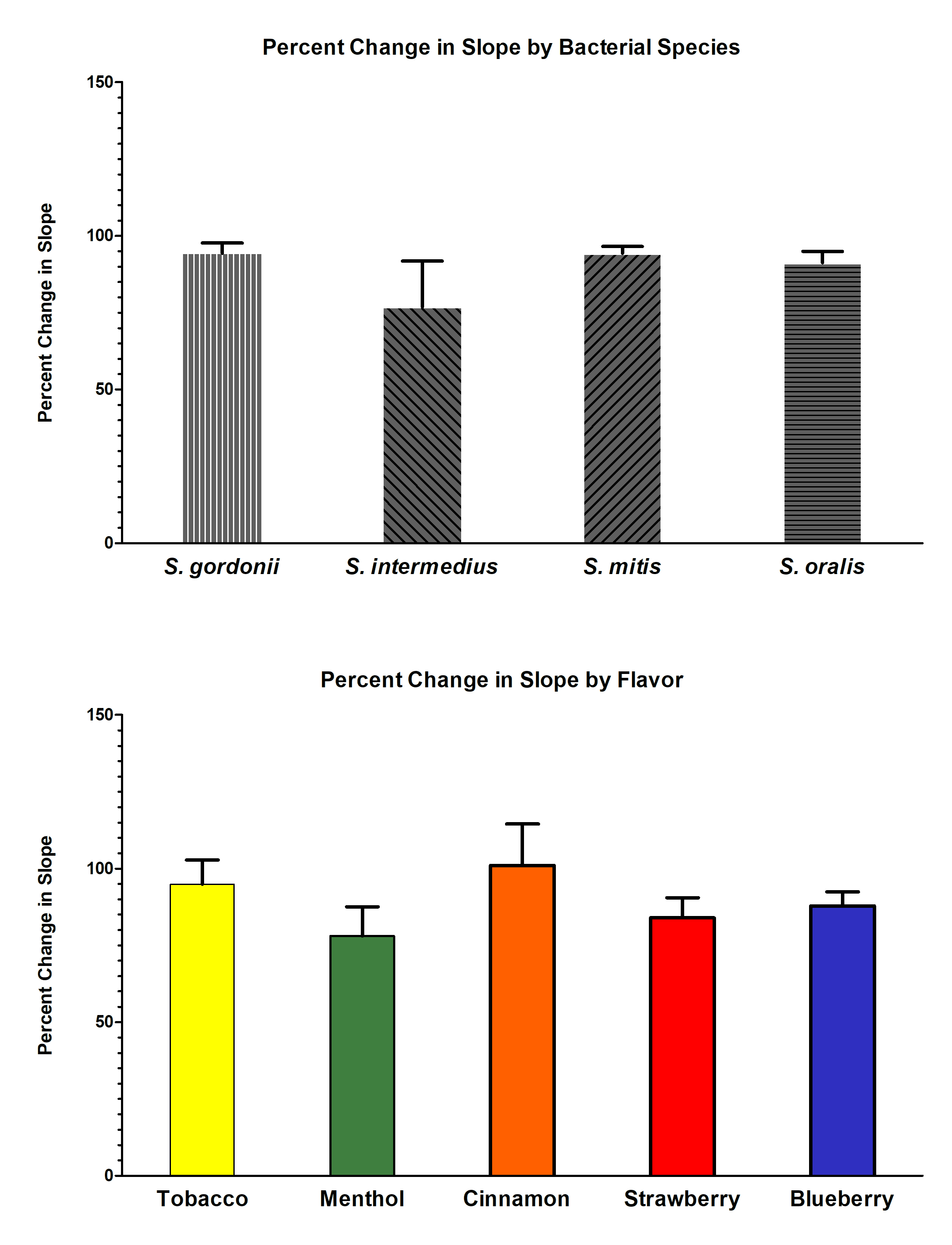

Supplement: Supplementary Figure 3 — Percent change in slope (combined data from Figures 4, 5) as compared to flavorless E-liquid; by bacterial species (upper panel) and by flavor (lower panel). Each bar represents mean ± SEM, n = 8 in upper panel and n = 10 in lower panel are the number of replicates. [file Image_3.TIF]

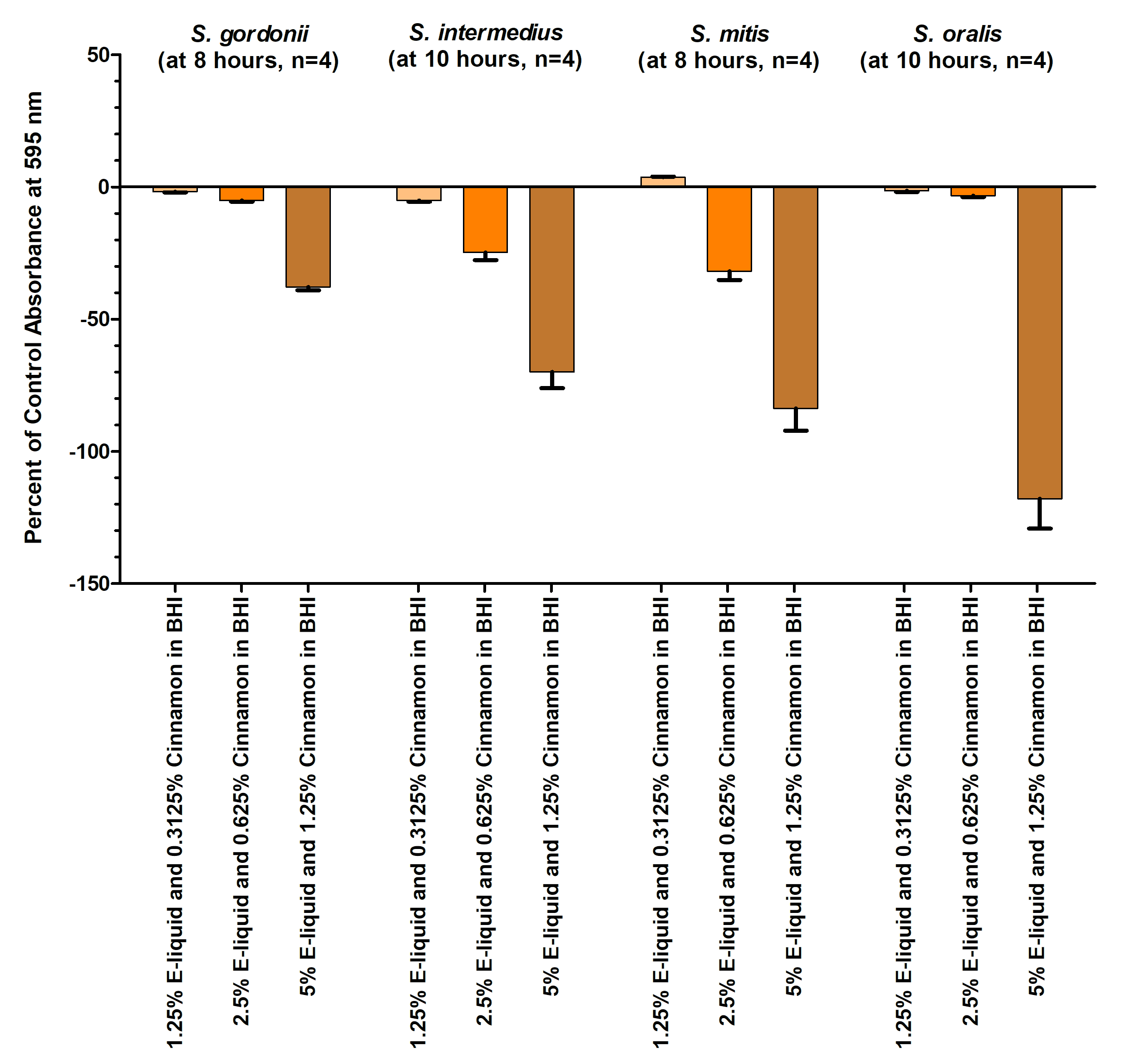

Supplement: Supplementary Figure 4 — Each bar represents mean ± SEM of the percent of control absorbance (OD 595) readings for all bacteria exposed to high concentration cinnamon flavor where n, as shown in the graph, is the number of replicates. [file Image_4.TIF]
