# Supplementary material for: Flavorless vs. Flavored Electronic Cigarette-Generated Aerosol and E-Liquid on the Growth of Common Oral Commensal Streptococci
Source: Front Physiol. 2020 Nov 23;11:585416. doi: 10.3389/fphys.2020.585416 (PMC7732452; doi:10.3389/fphys.2020.585416)
Supplement: Supplementary Table 2 — Complete list of statistics for Figure 7. [file Data_Sheet_1.PDF]

Supplemental Table 2: Complete List of Statistics from **Figure 7**.

	S.gordonii	***S.intermedius***	***S.mitis***	***S.oralis***
**Flavorless E-liquid**	Control vs 5% Hydrogen Peroxide, $p < 0.001$<br><br>1.25% E-liquid vs 5% Hydrogen Peroxide , $p < 0.001$<br><br>2.5% E-liquid vs 5% Hydrogen Peroxide , $p < 0.001$<br><br>5% E-liquid vs 5% Hydrogen Peroxide , $p < 0.01$	Control vs 5% Hydrogen Peroxide, $p < 0.01$<br><br>1.25% E-liquid vs 5% Hydrogen Peroxide , $p < 0.01$<br><br>2.5% E-liquid vs 5% Hydrogen Peroxide , $p < 0.05$	Control vs 5% Hydrogen Peroxide, $p < 0.001$<br><br>1.25% E-liquid vs 5% Hydrogen Peroxide , $p < 0.001$<br><br>2.5% E-liquid vs 5% Hydrogen Peroxide , $p < 0.001$<br><br>5% E-liquid vs 5% Hydrogen Peroxide , $p < 0.001$	Control vs 5% Hydrogen Peroxide, $p < 0.001$<br><br>1.25% E-liquid vs 5% Hydrogen Peroxide , $p < 0.001$<br><br>2.5% E-liquid vs 5% Hydrogen Peroxide , $p < 0.001$<br><br>5% E-liquid vs 5% Hydrogen Peroxide , $p < 0.01$
**E-liquid + Tobacco**	Control vs 5% Hydrogen Peroxide , $p < 0.001$<br><br>1.25% E-liquid (0.3125% Tob) vs 5% Hydrogen Peroxide , $p < 0.001$<br><br>2.5% E-liquid (0.625% Tob) vs 5% Hydrogen Peroxide , $p < 0.001$<br><br>5% E-liquid (1.25% Tob) vs 5% Hydrogen Peroxide, $p < 0.01$	Control vs 5% Hydrogen Peroxide , $p < 0.01$<br><br>1.25% E-liquid (0.3125% Tob) vs 5% Hydrogen Peroxide , $p < 0.05$<br><br>2.5% E-liquid (0.625% Tob) vs 5% Hydrogen Peroxide, $p < 0.05$	Control vs 5% Hydrogen Peroxide , $p < 0.001$<br><br>1.25% E-liquid (0.3125% Tob) vs 5% E-liquid (1.25% Tob), $p < 0.05$<br><br>1.25% E-liquid (0.3125% Tob) vs 5% Hydrogen Peroxide , $p < 0.001$<br><br>2.5% E-liquid (0.625% Tob) vs 5% Hydrogen Peroxide , $p < 0.001$<br><br>5% E-liquid (1.25% Tob) vs 5% Hydrogen Peroxide, $p < 0.01$	Control vs 5% E-liquid (1.25% Tob), $p < 0.05$<br><br>Control vs 5% Hydrogen Peroxide, $p < 0.001$<br><br>1.25% E-liquid (0.3125% Tob) vs 5% Hydrogen Peroxide, $p < 0.001$<br><br>2.5% E-liquid (0.625% Tob) vs 5% Hydrogen Peroxide , $p < 0.001$
**E-liquid + Menthol**	Control vs 5% E-liquid (1.25% Men), $p < 0.05$<br><br>Control vs 5% Hydrogen Peroxide, $p < 0.001$<br><br>1.25% E-liquid (0.0625% Men) vs 5% Hydrogen Peroxide, $p < 0.01$<br><br>2.5% E-liquid (0.125% Men) vs 5% Hydrogen Peroxide, $p < 0.01$	Control vs 5% Hydrogen Peroxide, $p < 0.01$<br><br>1.25% E-liquid (0.3125% Men) vs 5% Hydrogen Peroxide , $p < 0.05$	Control vs 5% Hydrogen Peroxide, $p < 0.001$<br><br>1.25% E-liquid (0.3125% Men) vs 5% E-liquid (1.25% Men), $p < 0.05$<br><br>1.25% E-liquid (0.3125% Men) vs 5% Hydrogen Peroxide, $p < 0.001$<br><br>2.5% E-liquid (0.125% Men) vs 5% Hydrogen Peroxide, $p < 0.001$	Control vs 5% E-liquid (1.25% Men), $p < 0.01$<br><br>Control vs 5% Hydrogen Peroxide, $p < 0.001$<br><br>1.25% E-liquid (0.3125% Men) vs 5% E-liquid (1.25% Men), $p < 0.05$<br><br>1.25% E-liquid (0.0625% Men) vs 5% Hydrogen Peroxide, $p < 0.01$

Supplemental Table 2: (continued).

	S.gordonii	*S.intermedius*	*S.mitis*	*S.oralis*
**E-liquid + Cinnamon**	Control vs 1.25% E-liquid (0.3125% Cin), $p < 0.01$<br>Control vs 2.5% E-liquid (0.625% Cin), $p < 0.05$<br>Control vs 5% Hydrogen Peroxide, $P < 0.001$<br>1.25% E-liquid (0.3125% Cin) vs 5% E-liquid (1.25% Cin), $p < 0.01$<br>2.5% E-liquid (0.625% Cin) vs 5% E-liquid (1.25% Cin), $p < 0.05$<br>5% E-liquid (1.25% Cin) vs 5% Hydrogen Peroxide, $p <0.001$	Control vs 5% E-liquid (1.25% Cin), $p < 0.01$<br>Control vs 5% Hydrogen Peroxide, $p < 0.01$<br>1.25% E-liquid (0.3125% Cin) vs 5% E-liquid (1.25% Cin), $p < 0.001$<br>2.5% E-liquid (0.625% Cin) vs 5% E-liquid (1.25% Peroxide), $p < 0.01$<br>2.5% E-liquid (0.625% Cin) vs 5% Hydrogen Peroxide, $p < 0.05$<br>5% E-liquid (1.25% Cin) vs 5% Hydrogen Peroxide, $p <0.001$	Control vs 1.25% E-liquid (0.3125% Cin), $p < 0.05$<br>Control vs 5% Hydrogen Peroxide, $p < 0.001$<br>1.25% E-liquid (0.3125% Cin) vs 2.5% E-liquid (0.625% Cin), $p < 0.05$<br>1.25% E-liquid (0.3125% Cin) vs 5% E-liquid (1.25% Cin), $p < 0.001$<br>2.5% E-liquid (0.625% Cin) vs 5% Hydrogen Peroxide, $p < 0.001$<br>5% E-liquid (1.25% Cin) vs 5% Hydrogen Peroxide, $p <0.001$	Control vs 1.25% E-liquid (0.3125% Cin), $p < 0.05$<br>Control vs 5% E-liquid (1.25% Cin), $p < 0.05$<br>Control vs 5% Hydrogen Peroxide, $P < 0.01$<br>1.25% E-liquid (0.3125% Cin) vs 5% E-liquid (1.25% Cin), $p < 0.001$<br>2.5% E-liquid (0.625% Cin) vs 5% E-liquid (1.25% Cin), $p < 0.001$<br>5% E-liquid (1.25% Cin) vs 5% Hydrogen Peroxide, $p <0.001$
**E-liquid + Strawberry**	Control vs 2.5% E-liquid (0.625% Str), $p < 0.01$<br>Control vs 5% E-liquid (1.25% Str), $p < 0.01$<br>Control vs 5% Hydrogen Peroxide, $p < 0.001$	Control vs 1.25% E-liquid (0.3125% Str), $p < 0.05$<br>Control vs 2.5% E-liquid (0.625% Str), $p < 0.05$<br>Control vs 5% E-liquid (1.25% Str), $p < 0.05$<br>Control vs 5%Hydrogen Peroxide, $p < 0.01$	Control vs 2.5% E-liquid (0.625% Str), $p < 0.05$<br>Control vs 5% E-liquid (1.25% Str), $p < 0.05$<br>Control vs 5%Hydrogen Peroxide, $p < 0.01$<br>1.25% E-liquid (0.31255% Str) vs 5% Hydrogen Peroxide, $p < 0.05$	Control vs 1.25% E-liquid (0.3125% Str), $p < 0.001$<br>Control vs 2.5% E-liquid (0.625% Str), $p < 0.001$<br>Control vs 5% E-liquid (1.25% Str), $p < 0.001$<br>Control vs 5%Hydrogen Peroxide, $p < 0.001$
**E-liquid + Blueberry**	Control vs 5% Hydrogen Peroxide, $p < 0.001$<br>1.25% E-liquid (0.3125% Blu) vs 5% Hydrogen Peroxide, $p < 0.001$<br>2.5% E-liquid (0.625% Blu) vs 5% Hydrogen Peroxide, $p < 0.001$<br>5% E-liquid (1.25% Blu) vs 5% Hydrogen Peroxide, $p < 0.01$	Control vs 5% Hydrogen Peroxide, $p < 0.01$<br>1.25% E-liquid (0.3125% Blu) vs 5% Hydrogen Peroxide, $p < 0.01$<br>2.5% E-liquid (0.625% Blu) vs 5% Hydrogen Peroxide, $p < 0.05$	Control vs 5% E-liquid (1.25 Blu), $p < 0.01$<br>Control vs 5% Hydrogen Peroxide, $p < 0.001$<br>1.25% E-liquid (0.3125% Blu) vs 5% Hydrogen Peroxide, $p < 0.05$<br>2.5% E-liquid (0.625% Blu) vs 5% Hydrogen Peroxide, $p < 0.001$<br>5% E-liquid (1.25% Blu) vs 5% Hydrogen Peroxide, $p < 0.01$	Control vs 5% E-liquid (1.25% Blu), $p < 0.01$<br>Control vs 5% Hydrogen Peroxide, $p < 0.001$<br>1.25% E-liquid (0.3125% Blu) vs 5% E-liquid (1.25% Blu), $p < 0.05$<br>1.25% E-liquid (0.3125% Blu) vs 5% Hydrogen Peroxide, $p < 0.001$<br>2.5% E-liquid (0.625% Blu) vs 5% Hydrogen Peroxide, $p < 0.01$
